# Human Spotted Fever Group *Rickettsia* Infecting Yaks (*Bos grunniens*) in the Qinghai-Tibetan Plateau Area

**DOI:** 10.3390/pathogens9040249

**Published:** 2020-03-28

**Authors:** Yingna Jian, Jixu Li, Paul Franck Adjou Moumouni, Xueyong Zhang, Maria Agnes Tumwebaze, Geping Wang, Qigang Cai, Xiuping Li, Guanghua Wang, Mingming Liu, Yongchang Li, Liqing Ma, Xuenan Xuan

**Affiliations:** 1 Qinghai Academy of Animal Sciences and Veterinary Medicine, Centre for Biomedicine and Infectious Diseases, Qinghai University, State Key Laboratory of Plateau Ecology and Agriculture, Xining 810016, China; jianyingna@hotmail.com (Y.J.); zhangxueyong86@hotmail.com (X.Z.); wanggeping001@outlook.com (G.W.); qgcai@hotmail.com (Q.C.); lixiuping1977@outlook.com (X.L.); wangguanghua2007@outlook.com (G.W.); 2 National Research Center for Protozoan Diseases, Obihiro University of Agriculture and Veterinary Medicine, Obihiro, Hokkaido 080-8555, Japan; JixuLi@hotmail.com (J.L.); chakirou82@yahoo.fr (P.F.A.M.); tumwebazeaggie@gmail.com (M.A.T.); lmm_2010@hotmail.com (M.L.); YongchangLi8762017@outlook.com (Y.L.)

**Keywords:** fever rickettsioses, human SFG *Rickettsia*, Qinghai-Tibetan Plateau, yak

## Abstract

The Qinghai-Tibetan Plateau Area (QTPA) is a plateau with the highest average altitude, located in Northwestern China. There is a risk for interspecies disease transmission, such as spotted fever rickettsioses. However, information on the molecular characteristics of the spotted fever group (SFG) *Rickettsia* spp. in the area is limited. This study performed screenings, and detected the DNA of human pathogen, SFG *Rickettsia* spp., with 11.3% (25/222) infection rates in yaks (*Bos* *grunniens*). BLASTn analysis revealed that the *Rickettsia* sequences obtained shared 94.3–100% identity with isolates of *Rickettsia* spp. from ticks in China. One *Rickettsia* sequence (MN536161) had 100% nucleotide identity to two *R. raoultii* isolates from Chinese *Homo sapiens*, and one isolate from Qinghai *Dermacentor* *silvarum*. Meanwhile, another *Rickettsia* sequence (MN536157) shared 99.1–99.5% identity to one isolate from *Dermacentor* spp. in China. Furthermore, the phylogenetic analysis of SFG *Rickettsia* spp. *ompA* gene revealed that these two sequences obtained from yaks in the present study grouped with the *R. slovaca* and *R. raoultii* clades with isolates identified from *Dermacentor* spp. and *Homo sapiens*. Our findings showed the first evidence of human pathogen DNA, SFG *Rickettsia* spp., from animals, in the QTPA.

## 1. Introduction

Rickettsioses are a known infectious diseases around the world, caused by obligate intracellular bacteria, the spotted fever group (SFG), *Rickettsia* [1,2,3]. Recently, the SFG *Rickettsia* species has caused diseases with varying clinical presentations in humans, domestic animals, and wildlife, and these diseases are considered an emerging global threat [1,4]. 

In China, eleven SFG *Rickettsia* species, *Rickettsia sibirica*, *Rickettsia heilongjiangensis*, *Rickettsia aeschlimannii*, *Rickettsia conorii*, *Rickettsia felis*, *Rickettsia massiliae*, *Rickettsia monacensis*, *Rickettsia rickettsia*, *Candidatus* Rickettsia jingxinensis, *Rickettsia raoultii* and *Rickettsia slovaca*, have been identified in tick vectors, domestic animals and wildlife [5,6,7,8,9,10,11,12,13]. Several *Rickettsia* genotypes have been characterized as etiological factors of human rickettsiosis in the country, such as *R. heilongjiangensis* [14,15], *R. sibirica *subsp. *sibirica BJ-90* [16], *R. raoultii* [17,18,19], *Rickettsia japonica* [20,21], *Rickettsia* sp. *XY99* [22], and *Candidatus* Rickettsia tarasevichiae [23]. These findings suggest that rickettsioses are emerging zoonoses in China.

The Qinghai-Tibetan Plateau Area (QTPA) is the largest plateau on the planet [24]. There is a special climate, comprised of a lower average annual temperature and rainfall that fluctuates, therefore, a variety of unique livestock are grazed there [25]. Among these livestock, the yak (*Bos grunniens*), which belongs to the bovine species, can survive in extreme environmental conditions, such as cold, harsh, and oxygen-poor [26]. The Qinghai plateau, located on the northeastern side of the QTPA, has a unique and vigorous natural ecosystem because of its high altitude, cold climate, and oxygen deficiency [27]. There is an abundant yak genetic resource with more than five million individuals in the Qinghai province [27]. Previous studies revealed that Qinghai was considered one of the origins and domestication locales for yaks based on mitochondrial DNA analyses [28]. 

Among the *Rickettsia* species, there are several important emerging pathogens in humans and animals. Although one study confirmed the infection of SFG *Rickettsia* in ticks from Qinghai province, northwestern China [27], information on the infection and molecular characteristics of these pathogens in humans and yaks in the province are still limited. Therefore, in this study, we screened yaks in Qinghai plateau for the existence of human pathogens and found that the DNA of *Rickettsia* was present in yak blood samples. 

## 2. Results

**Figure 1:**
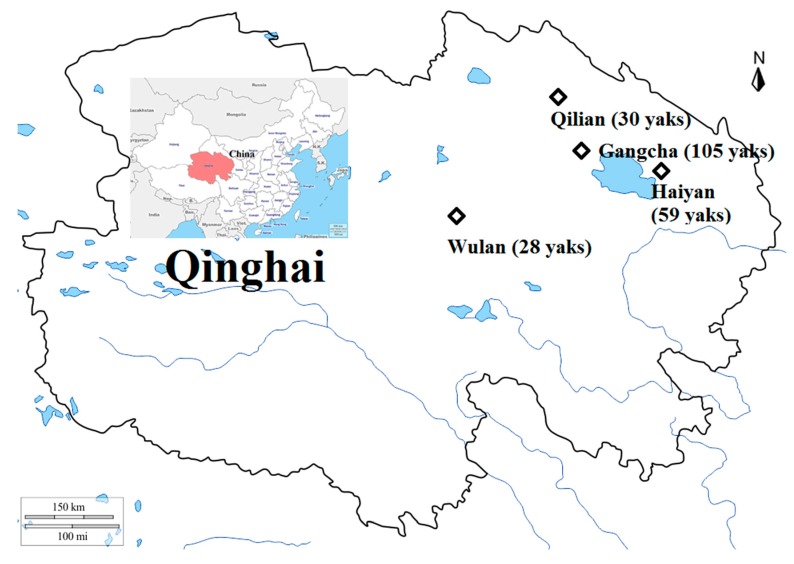
Map of Qinghai and China showing sampling areas and the number of animals indicated by the black quadrilateral. The map was generated using GIMP 2.8.10 (https://www. gimp.org).

In the present study, a total of 222 blood samples were collected from apparently healthy yaks in four counties on the Qinghai plateau, including Wulan (36°19′-37°20′ N and 97°01′-99°27′E, n = 28), Qilian (37°25′-39°05′N and 98°05′-101°02′E, n = 30), Haiyan (36°44′-37°39′N and 100°23′-101°20′E, n = 59), and Gangcha (36°58′-38°04′N and 99°20′-100°37′E, n = 105) (Figure 1). One pasture was chosen from each sampling site, and in each pasture, 10–20% of the stock was randomly selected for blood sampling from March to May 2018. Geographically, most of the livestock is raised in the northeastern pastoral region of the province. PCR screening based on outer membrane protein A (*ompA*) gene revealed that the overall infection rate was 11.3% (25/222) for SFG *Rickettsia* spp. (Table 1). Furthermore, from the four selected areas, the infection rates of SFG *Rickettsia* spp. in yaks are 10.7% (3/28) in Wulan, 13.3% (4/30) in Qilian, 5.1% (3/59) in Haiyan, and 14.3% (15/105) in Gangcha. The infection rate of SFG *Rickettsia* spp. in yaks from four areas was not significantly different (*p* > 0.05). 

Furthermore, a total of 25 SFG *Rickettsia* spp. sequences based on *ompA* gene were generated in the present study. Sequence analysis indicated that the SFG *Rickettsia* spp. infecting the yaks shared 94.3–100% identities to identified *R. raoultii* or *R. slovaca* isolates, as shown in Table 2. Meanwhile, SFG *Rickettsia* spp. sequences based on *ompA* gene were of three sizes (208, 209, and 212 bp). Additionally, BLASTn analysis of the *ompA* gene showed that the *Rickettsia* spp. obtained in this study had 85.4% to 100% identities to either sequence. Interestingly, one *Rickettsia* sequence (MN536161) obtained in the present study was 100% nucleotide identical to two *R. raoultii* isolates from Chinese *Homo sapiens* (JX945525 and KY474580), and three *R. raoultii* isolates from Chinese *Dermacentor* spp. (KF003015, KT899076 and MG228272) which include one isolate of *R. raoultii* from *Dermacentor silvarum* in Qinghai, China (MG228272). Furthermore, another *Rickettsia* spp. *ompA* sequence (MN536157) shared 99.5% identity to one *R. slovaca* isolate from *Dermacentor marginatus* (MF002535), and 99.1% identity to another *R. slovaca* isolate from *D. silvarum* (JN400402) in China. The five representative sequences obtained were submitted to GenBank and shown in Table 2.

**Figure 2:**
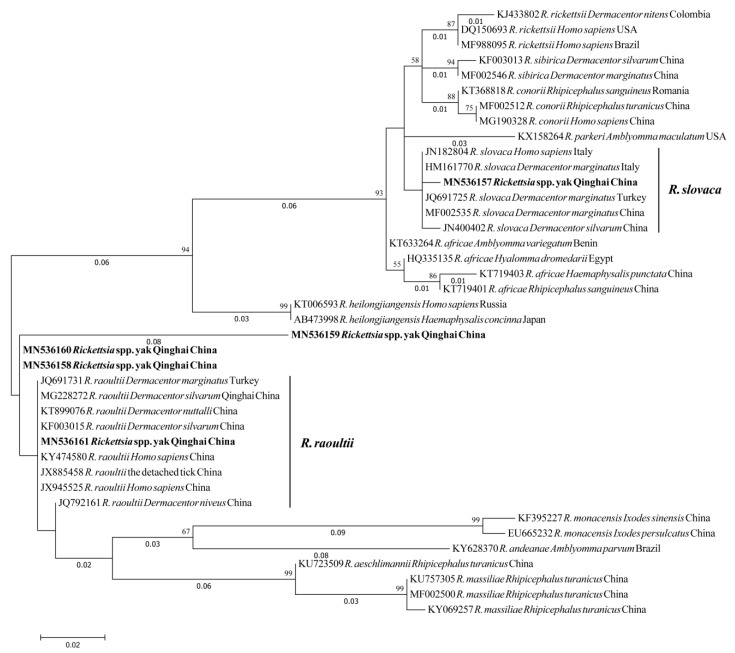
Molecular Phylogenetic analysis by Maximum Likelihood method based on *Rickettsia* spp. *ompA* partial sequences. Sequences from current study are marked in bold. The evolutionary history was inferred by using the Maximum Likelihood method based on the Kimura 2-parameter model. The tree with the highest log likelihood (−864.84) is shown. The percentage of trees in which the associated taxa clustered together is shown next to the branches. Initial tree(s) for the heuristic search were obtained automatically by applying Neighbor-Join and BioNJ algorithms to a matrix of pairwise distances estimated using the Maximum Composite Likelihood approach, and then selecting the topology with superior log likelihood value. A discrete Gamma distribution was used to model evolutionary rate differences among sites (5 categories (+G, parameter = 0.5571)). The tree is drawn to scale, with branch lengths measured in the number of substitutions per site (next to the branches). The analysis involved 40 nucleotide sequences. Codon positions included were 1st+2nd+3rd+Noncoding. All positions containing gaps and missing data were eliminated. There were a total of 186 positions in the final dataset. Evolutionary analyses were conducted in MEGA7.

The phylogenetic analysis of SFG *Rickettsia* spp. *ompA* gene revealed that two sequences obtained from yaks in the present study (MN536157 and MN536161) grouped with the *R. slovaca* and *R. raoultii* clades with isolates identified from *Dermacentor* spp. and *Homo sapiens* from China, Turkey, and Italy (Figure 2).

## 3. Discussion

This investigation reported the DNA detection of human *Rickettsia* species in the Qinghai-Tibetan Plateau Area. A previous study reported that SFG *rickettsiae*, including *R. raoultii, R. sibirica* subspecies sibirica, *Candidatus* R. tibetan, and *Candidatus* R. gannanii Y27 and F107 had a high overall infection rate in ticks from Qinghai, the sampling province of this study [27]. Our results document the first direct evidence of DNA detection of SFG *Rickettsia* pathogens in animals in this area. Importantly, sequencing analysis showed that two DNA sequences were 99.5% identity to *R. slovaca* and 100% nucleotide identical to *R. raoultii* isolates identified in China.

In China, *R. raoultii* infection cases with increasing numbers have been reported in humans [17,18,19,29]. These rickettsiose patients present not only lethargy, fever, and headache, but also neurological abnormalities [19]. Among these reports, a previous case which identified *R. raoultii* with 100% sequence identity between the isolate from patient and detached tick is very interesting [17], as the *R. raoultii* sequence obtained in this study also shared 100% identity to those previously identified from that patient (JX945525) and detached tick (JX885458). Furthermore, previous findings showed that *R. raoultii* was closely related with *Dermacentor* spp. [30]. The characterization of *R. raoultii* in yaks in the current study might indicate successful pathogen transmission from this tick species to yak, since one of the obtained *Rickettsia* sequences was 100% identical to those previously identified in *Dermacentor* spp. in Qinghai [27]. On the other hand, this *Rickettsia* sequence obtained also showed 100% identity with another isolate from human and four isolates from *Dermacentor* spp. from China. This suggests that humans and animals are both susceptible to this pathogen due to exposure to the same tick vectors.

*R. slovaca*, a member of human SFG rickettsiae, was first identified in *D. marginatus* in 1968 in Slovakia [31], and is now considered as the causative factor of tick-borne lymphadenopathy [32]. This human pathogen was reported for the first time in China in 2012 in *D. silvarum* ticks in Xinjiang province, which is the adjacent province to Qinghai [33]. In addition, *R. slovaca* was molecularly detected in shepherd blood DNA in Xinjiang Province [19]. However, compared to *R. raoultii* infections in humans and tick vectors in China, *R. slovaca* infection rate appears to be low and mild [17,33]. Previously, no reports have identified *R. slovaca* in ticks in Qinghai. In the present study, despite identifying only one yak positive, the DNA detection closely associated with *R. slovaca* provides evidence that this pathogen may exist in Qinghai.

In summary, this study found human pathogens, SFG *Rickettsia* spp., circulating in yaks in Qinghai of the Qinghai-Tibetan Plateau, China. The phylogenetic analysis of *ompA* gene revealed that sequences obtained from yaks in the present study were closely related to *R. slovaca* and *R. raoultii* isolates identified from *Dermacentor* spp. and *Homo sapiens*. Therefore, future studies are needed to provide data to the suspected importance of these ticks in transmitting the human pathogens to animals and humans in this plateau area.

## 4. Materials and Methods

Yak blood samples were collected into tubes, including anticoagulant (EDTA), and genomic DNA. The blood was extracted using and according to the manufacturer’s manual of the QIAamp DNA Blood Mini Kit (QIAGEN, Hilden, Germany). All yak DNA samples were screened with genus-specific primers (forward primer 5’–TGCGCCTTCGAGTTGTACAAGAG–3’ and reverse primer 5’–GACGGGTTGCRTAGGCTGAC–3’) based on *Rickettsia* spp. *ompA* gene [34]. The 10 μL PCR reaction mixture was used in the present study, containing 3 μL yak DNA template of blood, 0.1 μL Taq polymerase (0.5 U; New England BioLab, Ipswich, MA, USA), 0.5 μL each of forward and reverse primer (100 μM), 1 μL 10×ThermoPol Reaction Buffer (New England BioLab), 0.2 μL deoxyribonucleotide triphosphate (200 μM; New England BioLab), and double-distilled water up to 10 μL. Positive animal DNA samples from a previous study [8] were used as positive controls, and double-distilled water was used as a negative control.

All positive samples detected in this study were selected and used for sequencing analysis. The PCR products were purified using the QIAquick Gel Extraction Kit (QIAGEN), and then cloned into *E. coli* DH5α strain using the pGEM-T Easy Vector system (Promega, San Luis Obispo, CA, USA). At least three positive clones were selected to run sequencing by using Dye Terminator Cycle Sequencing Kit (Applied Biosystems, Foster City, CA, USA) by using ABI PRISM 3100 Genetic Analyzer (Applied Biosystems). The obtained sequences were confirmed by BLASTn search in GenBank, and then submitted to GenBank to get accession numbers. Phylogenetic trees were constructed using whole sequence fragment obtained by maximum likelihood statistical method with Kimura 2-parameter model and Gamma Distributed (G), and bootstrap analysis with 500 replications using MEGA7 [35]. 

The Chi-square test was performed to compare proportions of sample positivity in different sampling areas by Prism 7 software. Observed differences were considered to be statistically significant when *p*-values were <0.05.

All the procedures of the present study were accorded to the ethical guidelines of Obihiro University of Agriculture and Veterinary Medicine (280149). The protocol of the current study was also reviewed and approved by the Institutional Animal Care and Use Committee of the Qinghai Academy of Animal Sciences and Veterinary Medicine.

## Figures and Tables

**Table 1:** The infection rate of spotted fever group (SFG) *Rickettsia* in yaks.

*Rickettsia* spp.	Infection Rate (%) in Each Current Area				
	Wulan (n = 28)	Qilian (n = 30)	Haiyan (n = 59)	Gangcha (n = 105)	Total (n = 222)
Total positive	3 (10.7)	4 (13.3)	3 (5.1)	15 (14.3)	25 (11.3)
Negative samples	25 (89.3)	26 (86.7)	56 (94.9)	90 (85.7)	197 (88.7)

**Table 2:** DNA sequences obtained in this study.

Obtained DNA Sequence					The Cosest Blastn Match		
Pathogen	Gene	Accession Number	Length (bp)	Sequencing	Identity (%)	Species	Accession Number (Host, Country)
*Rickettsia* spp.	*ompA*	MN536157	212	*Rickettsia*	99.5	*R. slovaca*	MF002535 (tick, China)
		MN536158	209	*Rickettsia*	99.5	*R. raoultii*	MF511260 (tick, China)
		MN536159	208	*Rickettsia*	94.3	*R. raoultii*	MN450415 (tick, China)
		MN536160	209	*Rickettsia*	100	*R. raoultii*	MG811700 (tick, China)
		MN536161	209	*Rickettsia*	100	*R. raoultii*	KY474580 (human, China)
